# The color stability and wear resistance of provisional implant restorations: A prospective clinical study

**DOI:** 10.1002/cre2.311

**Published:** 2020-07-31

**Authors:** Vasilios Alevizakos, Gergo Mitov, Franziska Teichert, Constantin von See

**Affiliations:** ^1^ Center for Digital Technologies in Dentistry and CAD/CAM Danube Private University Krems an der Donau Austria; ^2^ Center for Prosthetic Dentistry and Dental Biomaterials Danube Private University Krems Austria

**Keywords:** abrasion, color, crown, implant

## Abstract

**Objectives:**

The aim of the present clinical study was to determine the stability of color and resistance against abrasion and attrition of provisional single implant restorations.

**Material and Methods:**

A group of 16 patients were treated with provisional crowns made of Telio CAD. Shortly before the insertion the crowns were photographed and scanned using a 3D‐laser scanner. After 8 weeks of clinical usage, the crowns were photographed and scanned again. The vertical occlusal wear and color changes between the restorations were measured.

**Results:**

The occlusal plane of the original crown showed a statistically significant reduction of 0.052 mm ± 0.037 mm 8 weeks after placement (*p* < .05). For the stability of color, a change in red, green and blue was described. All three scopes (red, green and blue) showed a statistically significant reduction (*p* < .05).

**Conclusions:**

This prospective clinical study showed that Telio CAD experienced a significant occlusal reduction and color change after an intraoral placement of 8 weeks.

## 1 INTRODUCTION

Over the past few decades, the reasons for visiting the dentist have dramatically changed. Apart of the treatment of pain and the functional rehabilitation of the jaw were in the foreground, nowadays dental treatment is focused on aesthetic optimization with a concomitant increase in self‐esteem and personal wellbeing (Deng, Wang, Deng, Liu, & Wu, 2018). In the case of missing teeth, there is a clear trend towards fixed restorations (Elagra, Alhayek, al‐Mutairi, Aljohar, & Aladwani, 2019). Accordingly, oral implantology plays an important role in today's dentistry when it comes to high quality replacement of missing teeth (Elagra et al., 2019).

Provisional crowns and bridges on natural posts and on implants help the patient to test aesthetic, phonetic and functional aspects before placing the permanent denture.

Thanks to digital technologies, it is now possible to produce such restorations via CAD/CAM processes (Mangano & Veronesi, 2018). After the digital design of a provisional crown or bridge, it is manufactured and provisionally inserted in the patient's mouth for testing. If the patient is satisfied with color, shape and wearing comfort, it is easy to mill exactly the same crown or bridge out of a definitive material. If desired, small and big changes can digitally be made, as long as the dentist and the patient have defined the individual optimum together. Nowadays research should focus in detail with provisional dental materials, their properties such as color variation and resistance, as well as wear behavior.

The aim of the present study was to determine the stability of color and resistance against abrasion and attrition of provisional single implant restorations.

## 2 MATERIAL AND METHOD

To address the research purpose, the investigators designed and implemented a clinical case study. A positive verdict by the Lower Austrian committee of ethics was obtained for our study. CAD/CAM fabricated PMMA blocks Telio CAD (Ivoclar Vivadent AG, Schaan, Liechtenstein) were used for the study. This material is a cross‐linked polymer block (PMMA) from which the monolithic crown can be ground out after scan and design. Due to the industrial production of the blanks and the subtractive manufacturing of the restorations a high homogeneity of the material is attained, and the polymerization shrinkage can be neglected.

The following inclusion and exclusion criteria have been established for the prospective clinical monocenter study with simple blinding:

### 2.1 Inclusion criteria

- Patient age: 25–85 years.
- Presence of an prothetically unrestored single‐tooth implant in the region of premolars or molars.
- Patient oth color in the spectrum of the PMMA blocks (A1‐3.5, B1, BL3).
- Antagonists present.

### 2.2 Exclusion criteria

- Poor oral hygiene.
- Severe marginal periodontitis.
- Nicotine consumption.
- Taking color‐changing medicines.

In the study, 14 patients were enrolled, whereby in one patient several implants with single tooth crowns could be placed. A total of 20 provisional implants was included in the study.

During the investigation two patients dropped out because of absence due to personal reasons.

A total of 16 implant crowns could be analyzed.

The starting point for the clinical study was always a single tooth gap treated with a BEGO implant (RSX‐Line, Semados, Bego, Bremen, Germany). The implants used for the study had diameters ranging from 4.1 to 4.5 mm. In the osseointegrated or primary stable single‐tooth implant, a scan post (CAD SP, Bego, Bremen, Germany) and a scan body (Omnicam, Sirona Dentsply, Bensheim, Germany) were inserted. The insertion was routinely checked with a single‐tooth X‐ray.

**FIGURE 1:**
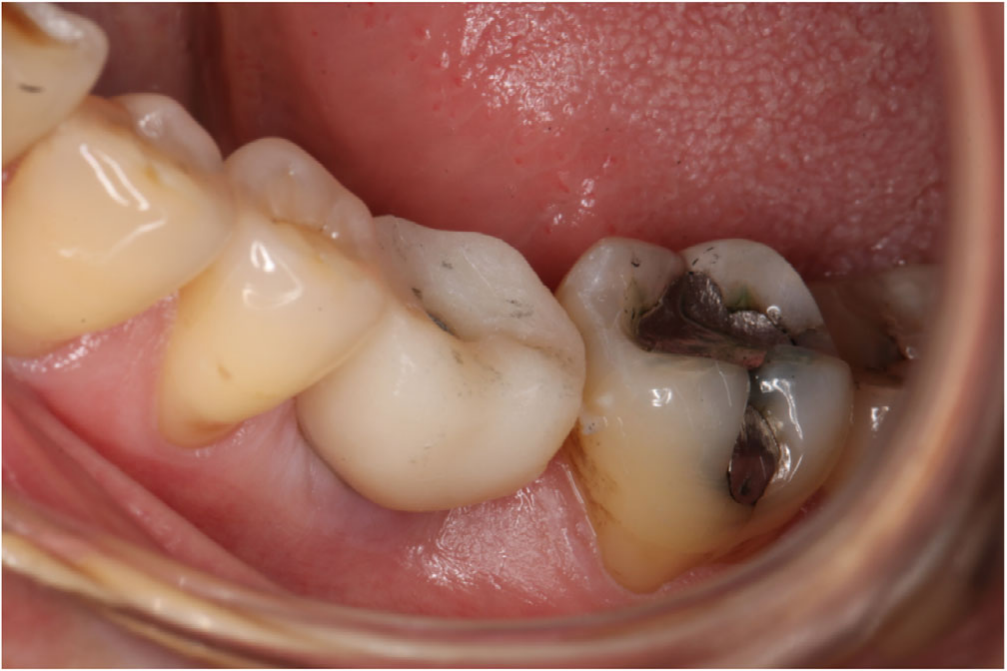
A crown being adjusted in the patient's mouth regarding occlusal and approximal contacts

The intraoral scan was performed using a Cerec Omnicam 3D‐scanner (Sirona Dentsply, Bensheim, Germany). According to the treatment protocol, the lower jaw, upper jaw, buccal bite and bite registration were recorded here. The Telio CAD Abutment Solution crowns on single‐tooth implants were produced using a CAD software (Cerec SW 4.4.4.) and a chairside milling unit (Cerec MCXL, Sirona Densply, Bensheim, Germany). After milling the crown was post processed and glued (Multilink Hybridabutment, Ivoclar Vivadent AG, Schaan, Liechtenstein) to the titanium base (PS TiB, Bego, Bremen, Germany). The crowns were adjusted in the patient's mouth regarding occlusal and approximal contacts (Figure 1). The finishing of the crown was performed by manual polishing with pumice.

**FIGURE 2:**
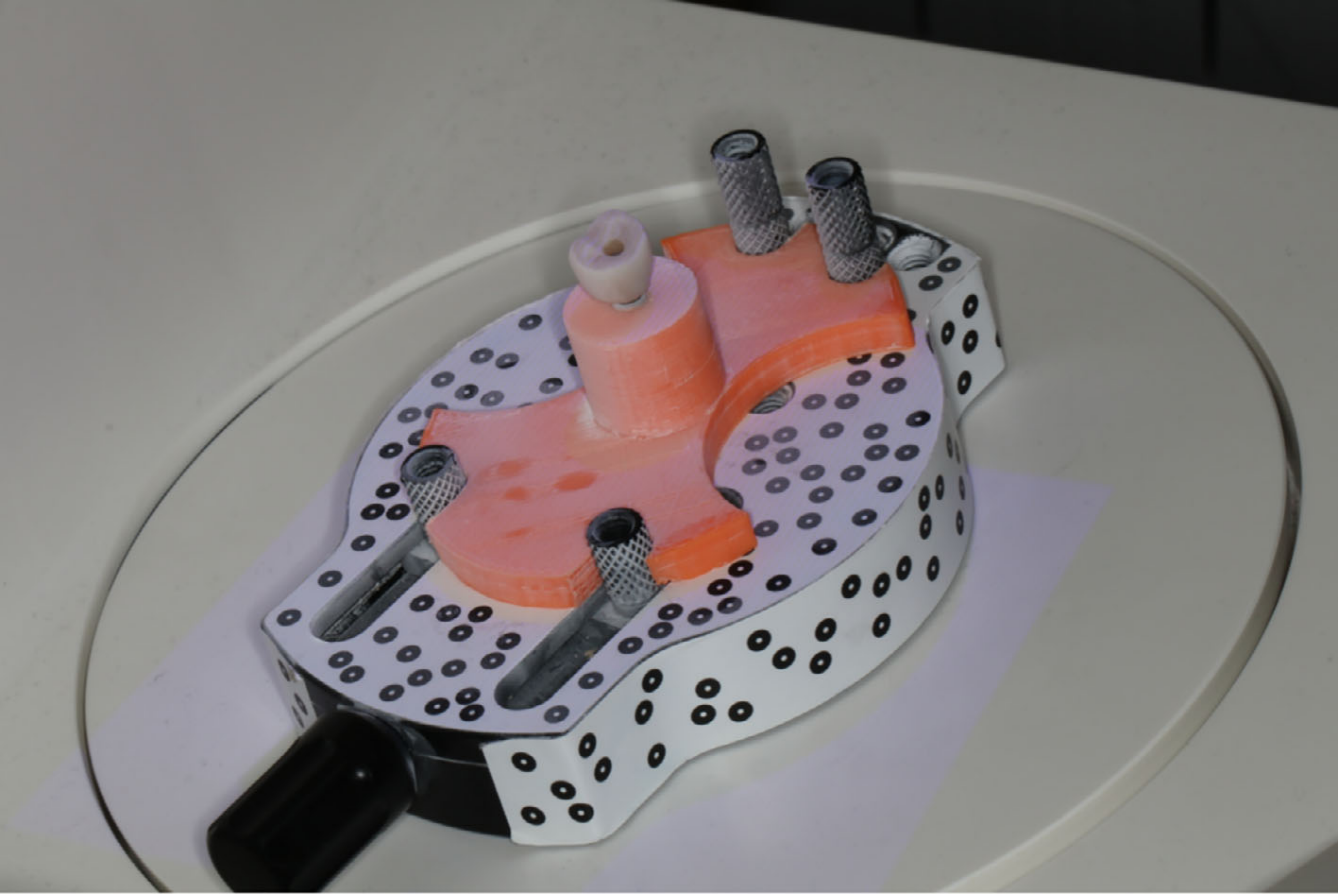
The customized crown‐holder fixed in the Lab scanner

Before insertion the crowns were digitally recorded with a 3D laser scanner (Aadva Lab Scan, GC, Leuven, Belgium). In order to ensure a reproducible position of the crown in the fully automated laboratory scanner, a special holder was 3D‐printed (Figure 2). Prior to the scan matting of the implant crown surface was necessary to allow adequate scanning of the entire surface. Therefore, a matting 3D‐scan powder spray was used. Reflections on the blue LED light would produce holes in the surface representation and then lead to faulty evaluation with the 3D software. The provisionals crown were scanned with the laboratory scanner once before and once after the eight‐week wearing period and the data saved as .stl files.

To record any color differences after a period of 8 weeks of clinical wear, digital initial and final photographs were taken under conditions that were as constant as possible. For this purpose, the provisional crowns were photographed with a single‐lens reflex camera

(Canon EOS 80D) after the polishing and after the digital data acquisition. Care was taken to ensure that the crown was again cleaned under running water and soap after being scanned with the laboratory scanner and then dried with a clean paper towel. Under the same conditions a photo was taken after the 8‐week period.

When selecting the measuring points, care was taken to never measure in the area of the reflection of the ring flash, the strongest curvature, and also in the area below the equator, since the silver‐colored titanium base passing through them would lead to different measurement results.

As soon as the .stl files of the scanned crowns were available, surface analysis was performed using a evaluation software (Inspect 8 SR 1, Gom GmbH, Braunschweig, Germany).

**FIGURE 3:**
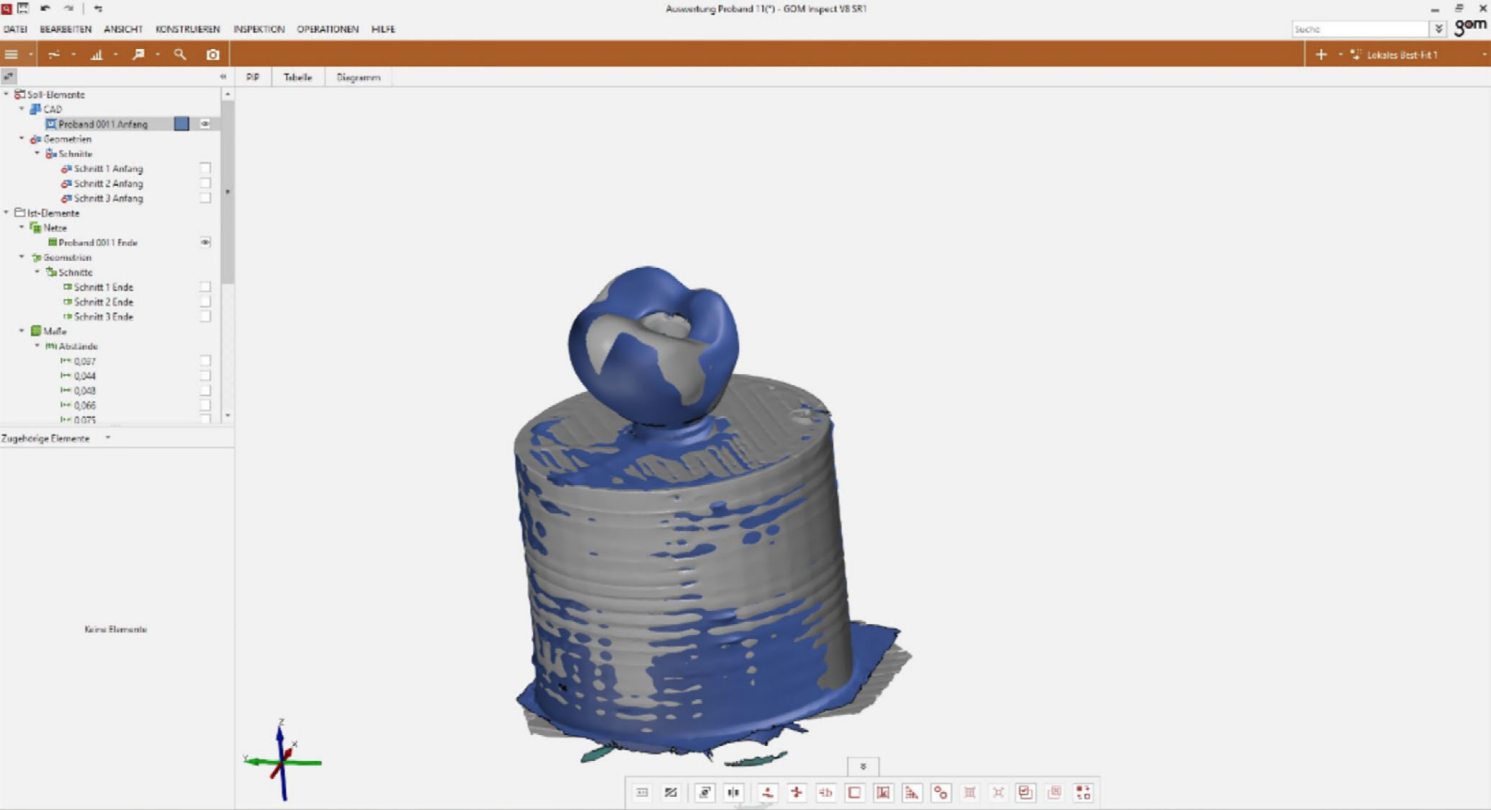
Matching of both datasets—prior and after 8 weeks of loading

**FIGURE 4:**
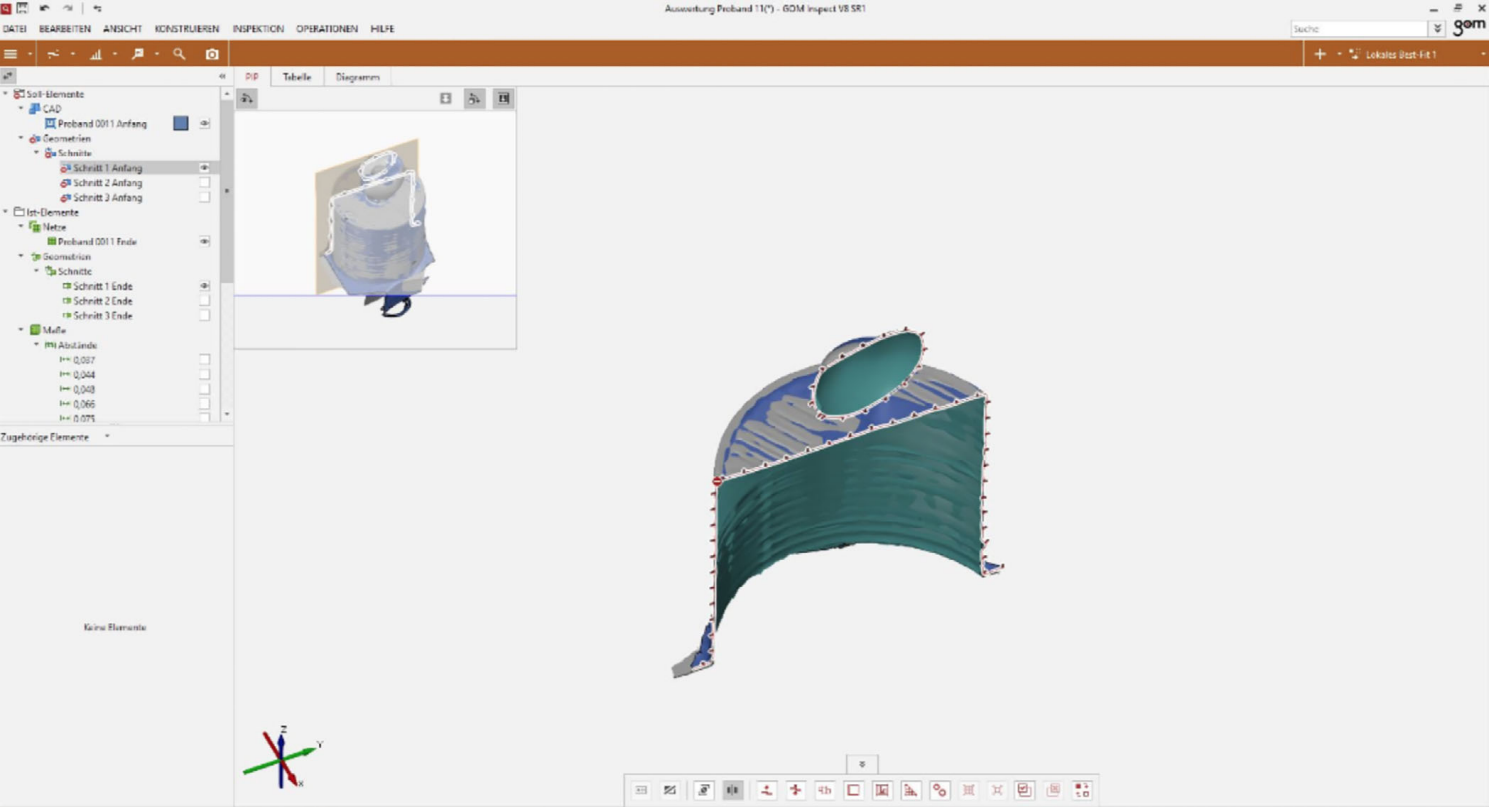
Slicing the dataset‐compound for analysis

After overlaying the initial dataset with the final dataset (Figure 3), three arbitrary slice planes were defined which were identical for both datasets (Figure 4). Subsequently, the cutting planes were superimposed, so that the distances between the “before” and “after” planes could be visualized with a two‐point measurement. Since five locations per sections were analyzed, a total of 15 measured values were available for the statistical analysis.

**FIGURE 5:**
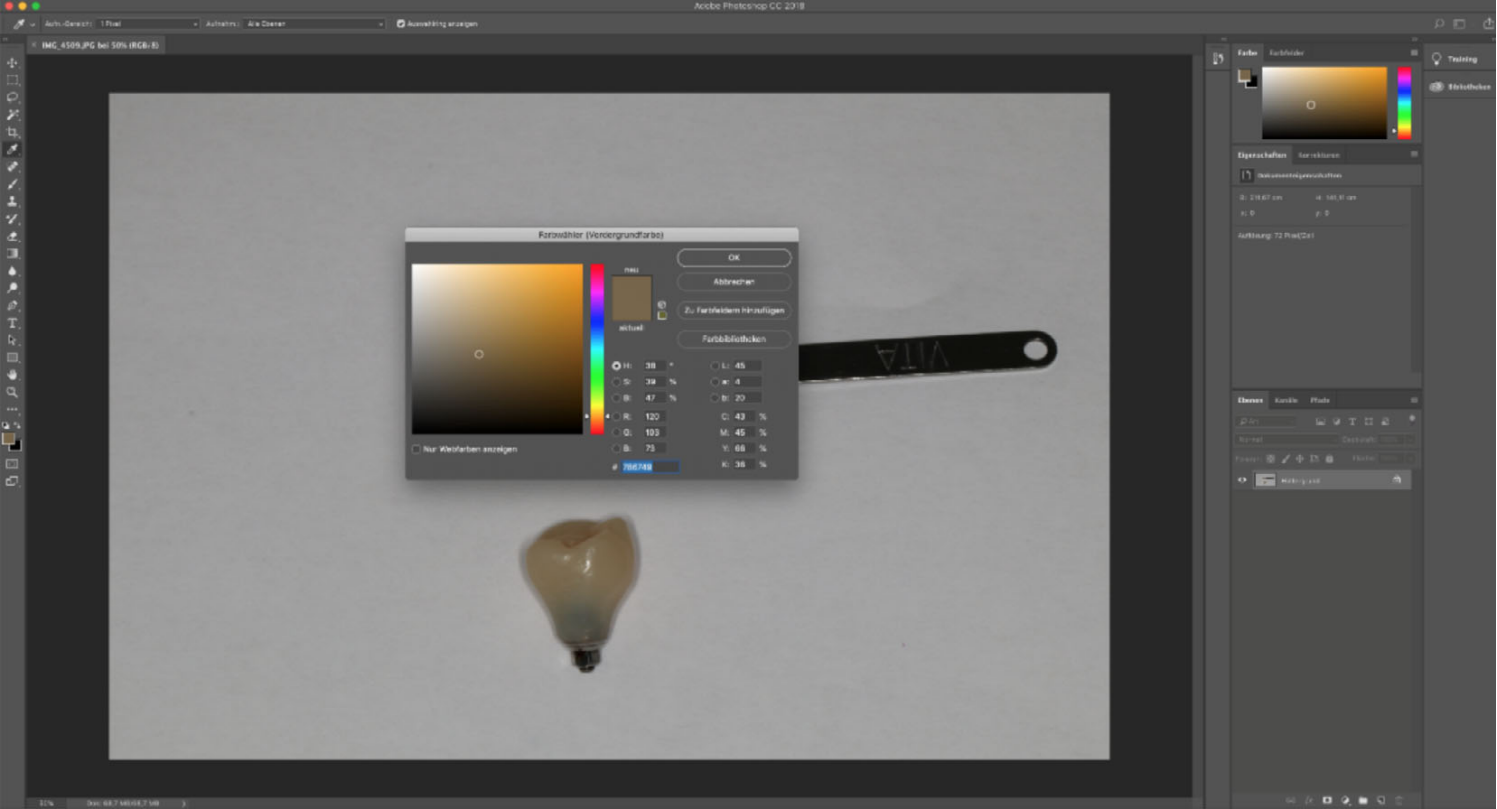
Colorimetry: RGB‐value analysis

For the evaluation of color stability, a purely visual representation of color changes through photography was omitted in this clinical study. The reliable comparison was enabled by the collection of RGB values in initial and final photographs for each crown (Figure 5).

RGB represents the amount of the individual colors red, green and blue. The individual components were analyzed with the photo [RAW] software Photoshop (CC 2018, Adobe, San Jose, CA). Five points were randomly chosen and evaluated according to RGB.

A total of 240 measured values were included in the statistical evaluation of the occlusal wear.

A total of 480 readings were included in the statistical evaluation of the color stability. First, the available data was checked with the Kolmogorov–Smirnov adaption test. Differences between the groups were tested with the Wilcoxon test because of the missing normal distribution. The statistical significance level was set at *p* = .05.

## 3 RESULTS

### 3.1 Wear resistance

The smallest abrasion was 0.003 mm, while 0.23 mm was the maximum surface reduction. The mean value was of 0.052 mm ± 0.037 mm. A high statistical significance height reduction of the provisional crowns could be registered (*p* < .001).

### 3.2 Color stability

The intraoral stay caused colour changes. In the groupings red, green and blue, there was a statistically significant reduction for the measured color components (*p* < .05). In the mean value the amount of the primary color red decreased from 117.49 ± 10.2 to 115.10 ± 9.26, the amount of the basic color green from 99.08 ± 10.71 to 97.21 ± 10.41 and the amount of the primary color blue from 69.14 ± 10.66 to 67.56 ± 10.16.

## 4 DISCUSSION

### 4.1 Results

The present clinical trial confirmed a slight attrition of PMMA hybrid abutment crowns after a period of 8 weeks. With average wear of 0.052 mm ± 0.037 mm with statistical significance (*p* < .05), these results are consistent with the existing literature (Wimmer, Huffmann, Eichberger, Schmidlin, & Stawarczyk, 2016).

After all, not only natural teeth are exposed to physiological and pathological abrasion, but also dental materials. Even ceramic crowns and bridges may lose their occlusal height over the course of the wearing period, but they are the primary cause of occlusal loss of hard tooth tissue in natural anterior teeth (Suputtamongkol, Anusavice, Suchatlampong, Sithiamnuai, & Tulapornchai, 2008). Due to the inferior mechanical properties, it can be assumed that provisional materials do not have the same abrasion resistance as the natural enamel or ceramic restorations (Jang et al., 2019). For example, Ivoclar Vivadent AG specifies a flexural strength of 135 MPa for its provisional PMMA material Telio CAD, while the flexural strength of the glass–ceramic IPS e.max CAD is 530 MPa. Habib et al. demonstrated that after 240,000 chewing cycles, PMMA materials achieve a higher level of tooth enamel abrasion than zirconium oxide or veneering ceramics (Habib, Alotaibi, al Hazza, Allam, & AlGhazi, 2019). In its function as a short‐term provisional restoration, a comparable abrasion stability to the ceramic materials is not essential. However, some studies have shown that the stability and bending strength of many provisional materials, especially those of the industrially manufactured CAD/CAM blocks, are no longer affected by the average chewing activity and can therefore be used without hesitation as long‐term provisional restoration (Stawarczyk, Schmutz, Fischer, & Hammerle, 2010; Stawarczyk, Trottmann, & Fischer, 2008). Stawarczyk et al. even showed that the PMMA material Telio CAD from Ivoclar Vivadent AG has comparable wear resistance to glass ceramic materials (Stawarczyk et al., 2013). The annual wear rate of molar enamel in non‐bruxers is reportedly very low (29 μm), though a high biting force and parafunctional habits such as bruxism can accelerate tooth wear (Lambrechts, Braem, Vuylsteke‐Wauters, & Vanherle, 1989). For patients with bruxism some studies have shown that these values can rise to 720–815 N (Pihut, Wisniewska, Majewski, Gronkiewicz, & Majewski, 2009; Tortopidis, Lyons, Baxendale, & Gilmour, 1998). In the study of Karaokutan et al. two of their PMMA groups (Temdent and Takilon) fractured within these values (Karaokutan, Sayin, & Kara, 2015). However, for patients with bruxism, clinicians should choose the temporary crown and bridge material with care to avoid failures.

Although this study demonstrates statistically significant abrasion after 8 weeks of intraoral wear, the clinical relevance of these results is questionable. With abrasions of 0.052 mm ± 0.037 mm after 8 weeks of wearing, it is not to be expected that the patient perceives a change in the bite position or a clinical relevant elongation of the antagonists can occur. It has been shown that the human sensory system can perceive changes in the occlusion from a hair thickness (Port & Euler, 2013). Assuming that a hair is about 0.1–0.8 mm thick, the measurement results with the mean value of 0.052 mm ± 0.037 mm are significantly lower.

Regarding the color stability, it was possible to objectively detect the change in the RGB values after an 8‐week wearing period of the Telio CAD Abutment Solution crowns. The content of the primary color red decreased statistically significantly from 117.49 ± 10.2 to 115.10 ± 9.26, the content of the primary color green from 99.08 ± 10.71 to 97.21 ± 10.41 and the content of the primary color blue from 69.14 ± 10.66 to 67.56 ± 10.16. In‐vitro studies showed that coffee and red wine in particular lead to statistically significant color changes after 7 days, depending on the BisGMA content of the materials with a lower extrinsic discoloration of PMMA with an increasing BisGMA content (Asmussen, 1986; Ítalo Alisson da Fonsêca & Emilena Maria Castor Xisto, 2018). Further studies proved that PMMA tend to change color after 15 days storage in turmeric solution, coffee, tea and Pepsi. In addition, it was shown that the discoloration was slightly lower with increased BisGMA content (Gupta & Gupta, 2011). Since the material Telio CAD does not consist of BisGMA, but consists of 95% polymethyl methacrylate, it can be assumed that the discoloration is lower here.

The fact that not only the BisGMA content but also the performance of an adequate polish can lead to increased color stability has also been discussed in in vitro studies (Rutkunas, Sabaliauskas, & Mizutani, 2010). Arocha et al. studied the color stability of CAD/CAM–PMMA blocks compared to laboratory produced provisional restorations. After a 4‐week storage of both types of PMMA in coffee, wine and tea, it could be shown that the CAD/CAM blocks have a statistically significantly higher discoloration (Arocha et al., 2014).

A comparison of the present clinical trial with the above mentioned is only very limited possible. The studies indicated are in vitro tests in which the test bodies were permanently exposed to the respective solutions coffee, red wine, cola and turmeric. As a control group, a saliva‐like substance was used in principle. The provisional crowns located in the oral cavity were only wetted by their own saliva apart from food intake, which is why there is overall less discoloration than in these in vitro studies.

### 4.2 Materials

The surface quality is of essential importance for the wear behavior and color stability of dental materials. The final polishing after the digital acquisition by means of laboratory scanner was always carried out manually. According to the recommendations of Ivoclar Vivadent AG, manual polishing with “moderate contact force” is sufficient to achieve the promised high color stability. Although the crown has always worked the same person with the same materials (pumice paste and diamond polishing paste) for 15 seconds at 10,000 rpm, an inaccuracy due to the manoeuvrability and the associated different contact pressure cannot be ruled out.

### 4.3 Methods

The abrasion after an average of 0.052 mm ± 0.037 mm after 8 weeks is significantly higher than the detection accuracy of the laboratory scanner Aadva Lab from the company GC (0.01 mm). Nevertheless, inaccuracies in the methodology must be assumed.

For the digital data acquisition and the production of a STL‐file, the adapted and highly polished crown was scanned with the GC Aadva Lab Scanner. The matting process may have an influence on the measurement results. Although Lee et al. have shown in their in vitro study that the precision of three‐dimensional scanners is generally getting better, loading the surface with matting spray can lead to slightly larger three‐dimensional changes. The scan deviations from a prefabricated master model in their study were 7.10 μm without powder and 8.65 μm with powder (Lee et al., 2016). Here, however, the question arises as to whether this difference is relevant in practice or can be avoided. In the case of a scan deviation without powder, the laser scanner itself can be made responsible for the deviation, since a scanning of the surface due to reflections is very difficult.

It is also very likely that the lab scanner itself has limited precision. The company GC specifies an accuracy of 10 μm for its Aadva Lab scanner (GC Europe N.V. 2016). Applying a matting powder, as recommended by the manufacturer, will make the surface detection easier for the scanner, but user errors during spraying cannot be avoided. Even by the same spray distance, a reproducible layer thickness of the powder on the provisional crown cannot be guaranteed (Todorović, Lisjak, Lazić, & Spadijer‐Gostovic, 2010). If the layer thickness of the matting is more than 10 μm, the deviation is more the accuracy of the GC Aadva laboratory scanner (scan‐accuracy: <10 μm).

After three‐dimensional coverage of the plastic crowns before and after the 8‐week time in situ with the GC Aadva Lab Scanner, the created STL‐files could be imported into a 3D survey software (GOM Inspect, version 8 SR 1). This analysis software for 3D data is used by industry as a standard for analysis or measurement and, although not necessarily in the dental field, is also used in one or the other studies (Seidel, Schmitt, Bergauer, Wichmann, & Matta, 2018; Szymor, Kozakiewicz, & Olszewski, 2016).

All provisional crowns were photographed with a Canon EOS 80D after the high gloss polish under conditions that were as constant as possible. Under the same conditions, these crowns were recaptured after the eight‐week wearing period. Although the photographies of the same regions were taken from the same object‐camera distance and angle and under constant light conditions, errors in reproduction cannot be completely ruled out.

For the quantitative data collection, the RGB color space and Photoshop (CC 2018) photo software were used in this clinical study. The RGB color space is often used for the representation of colors in the area of technical display devices such as computer monitors (International Lighting Commission 2019). In each case, the content of the primary colors red, green and blue is determined, which ultimately forms a new color. All values close to zero give the color black, all values against 255 are considered as completely additive mixed and give the color white.

Many surveys did not use digital photography with the RGB color space to compile the measurements, but rather a somewhat learning‐intensive, more elaborate and costly method. With the help of a colorimeter or spectrophotometer, objective measurements can be recorded within the framework of instrumental colorimetry, and even very small color differences, which are no longer perceptible to the human eye, can be quantitatively represented (Ergun, Mutlu‐Sagesen, Ozkan, Demirel, 2005; Haselton, Diaz‐Arnold, & Dawson, 2004; Scotti, Mascellani, & Forniti, 1997; Turker, Kocak, & Aktepe, 2006).

Nevertheless, the color representation via the RGB color is used in addition to all other color spaces such as CIELab, HSV (Hue, Saturation, Value) and HSL (Hue, Saturation, Lightness; Le, Swiatek, & Nguyen, 2010). However, other studies have demonstrated the sufficient apply of the RGB color space to compile measurements. Laskarin et al. uses the RGB values to analyze the influence of age on the tooth root color changes (Laskarin, Brkić, Pichler, & Buković, 2006). In the in vitro analysis of Kouadio et al. the staining effect of various chlorhexidine‐based mouthwashes was investigated. For quantifying the staining the RGB values were used (Kouadio et al., 2017). Furthermore, the study Carney and Johnston aimed to correlate RGB data from the VITA Linearguide 3D Master and VITA Bleached Guide 3D Master shade guides with their spectroradiometric correlates through a regression model while indicating a methodology for validation of accuracy of digital imaging systems (Carney & Johnston, 2016). They developed regressions models that allow tooth color information to be translated from digital images to accurate shade tab correlates for color matching purposes in dentistry (Carney & Johnston, 2016).

Since the dental practice already uses a lot of SLR cameras to document patient cases, their use can be extended and the exact determination of their tooth shades and their changes can be made with the aid of intuitive photo software and the RGB system.

Since only 16 subjects were available in the study and the wearing period of 8 weeks is significantly shorter than the maximum permitted 12 months, the data should be interpreted with caution.

## 5 CONCLUSION

Within the limitations of this study, the following conclusions can be drawn:

- The material Telio CAD Abutment Solution from Ivoclar Vivadent AG objectively has a statistically significant surface reduction after an intraoral 8‐week wearing period, but its clinical relevance should be called into question.
- The material Telio CAD Abutment Solution from Ivoclar Vivadent AG objectively has a statistically significant color change after an intraoral 8‐week wearing period, but its clinical relevance should be questioned.

## CONFLICT OF INTEREST

We declare that there are no conflicts of interest.

## ETHICS STATEMENT

Received (GS1‐EK‐4/387‐2016).
